# The pros and cons of multiple puncture in percutaneous balloon compression for treatment of trigeminal neuralgia

**DOI:** 10.3389/fneur.2022.1034133

**Published:** 2022-10-17

**Authors:** Chenglong Sun, Wenhao Zheng, Qiang Zhu, Quan Du, Wenhua Yu

**Affiliations:** ^1^Department of Neurosurgery, Affiliated Hangzhou First People's Hospital, Zhejiang University School of Medicine, Hangzhou, China; ^2^Department of Neurosurgery, Hangzhou Ninth People's Hospital, Hangzhou, China

**Keywords:** percutaneous balloon compression, repuncture, trigeminal neuralgia, repuncture methods, pain

## Abstract

**Background:**

Percutaneous balloon compression (PBC) is an effective and well-established surgery for treating trigeminal neuralgia (TN). However, if the initial attempt fails to produce a distinct pear shape, there is no conventional strategy to follow: repeat a few days later or re-puncture?

**Aims:**

This study aimed to analyze the risk and gain of re-puncturation in PBC surgery for TN treatment.

**Methods:**

We reviewed radiographs and medical records from 79 consecutive PBC cases. The complications and surgical outcomes were compared between one-time success pears and multiple re-puncturing pears. Re-puncturing methods included selecting a more appropriate entry point, a more possible entry angle, finding a stretchy spot around the margin of foramen ovale (FO) with a trocar, and exploring the direction with more resistance using a thinner guiding needle.

**Results:**

In 50% of cases, satisfactory pears were obtained after the first puncture, and in 35% of cases, satisfactory pears were obtained following re-puncturation. Except for hemihypogeusia, which was significantly more in multiple punctures cases (*p* < 0.05), no additional adverse effects were statistically different between the two groups. There are very few rare complications associated with re-puncturation. Log-Rank test of pain-free rate revealed no statistically significant differences between the two groups (*p* = 0.129).

**Conclusion:**

This study establishes the safety of re-puncturation in PBC surgery for TN treatment. The operation increases pears and does not cause any serious complications. The surgical outcomes of re-puncturation pears are almost identical to those one-time success pears.

## Introduction

Percutaneous balloon compression (PBC) is an effective therapeutic technique for trigeminal neuralgia (TN). In the 1980's, Mullan first introduced and described the percutaneous procedure (1, 2). The treatment modality remains popular nowadays due to its low cost and relative simplicity.

Although a pear-shaped balloon has been considered the gold standard for surgical success since its inception (2–6), the operator may quite often encounter non-optimal pear shapes. To repeat a few days later or re-puncture is a binary choice that many new practitioners of this method may struggle with. Konstantinos suggested that “persistent elliptical balloon shapes should raise consideration of aborting the procedure (5).” However, Asplund's policy was “not to immediately reoperate on a patient if the alleged optimal pear shape was not observed intraoperatively (3).” So far, there is no consensus on this aspect of the procedure.

Does re-puncturation increase the risk rate of postoperative complications? After acquiring an incorrect pear, how many times could the operator attempt before considering aborting the procedure? Previous research has yielded no conclusive solutions to these questions. By analyzing our series, we were able to address these questions. This study aimed to determine the risk and gain of re-puncturation in the outcome in patients treated with percutaneous balloon compression.

## Methods

### Patient population

Between May 2017 and January 2019, 79 patients underwent PBC operations. The patients in this series were diagnosed with TN, and PBC candidates have typical TN pain characteristics such as intense, electric shock with trigger point, sporadic, a positive response to carbamazepine (early stage), etc. They failed medical therapy and were at a high risk of undertaking a microvascular decompression (MVD) or were still experiencing pain following MVD. Table 1 summarizes the characteristics of cases.

**Table 1:** Characteristics of 79 patients undergoing PBCs.

**Characteristics**	**Patients (*n* = 79)**
Sex (No. of male/No. of female)	30/49
Median age at treatment [years (range)]	72 (31–91)
**Branch of pain**	
V1	4 (5.1)
V2	29 (36.7)
V3	22 (27.8)
V1+V2	6 (7.6)
V1+V3	1 (1.3)
V2+V3	13 (16.5)
V1+V2+V3	4 (5.1)
**Previous surgery for TN**	
Craniotomy (MVD, CPA tumor)	31 (39.2)
Minimal invasive techniques	12 (15.2)
Multiple treatments	4 (5.1)
None	32 (40.5)

Data presented as %, unless otherwise indicated.

V1, V2, V3 = first, second, and third branches of the trigeminal nerve.

Minimally invasive techniques: PBC, glycerol rhizotomy, electrocoagulation, gamma knife.

Multiple treatments = craniotomy + minimally invasive techniques.

### Puncturing technique

Under general anesthesia, the patient is placed in neutral supine position with head on a radiolucent headrest. After general anesthesia, lateral projections of patients were obtained using a C-arm fluoroscope. The patients' position and C-arm were kept still during the whole process.

**Figure 1:**
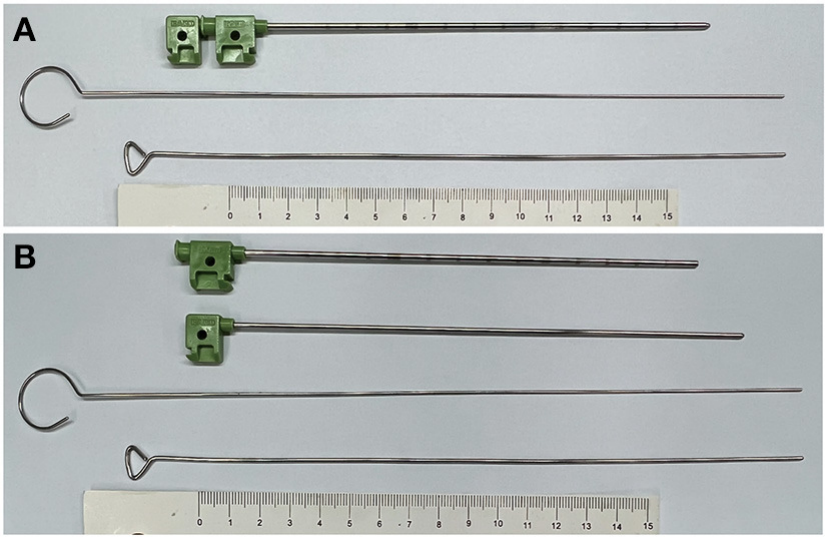
Puncture kits. **(A)** PBC puncture kits. From top to bottom: head-blunted liver-biopsy needle with stylet in, thinner needle with a blunt head, needle with a wider diameter, ruler (for scale). **(B)** PBC puncture kits with the stylet out.

We used a blunt-head trocar to percutaneously create a tunnel. Additional cannulations were performed using two longer needles of varying diameters (Figures 1A,B). Härtel's pathway was utilized to perform the puncture, but we slightly lifted the end (not the tip) of needle to increase the entry angle. The process followed De Cordoba et al.'s description (proposed by Henderson) (7, 8).

**Figure 2:**
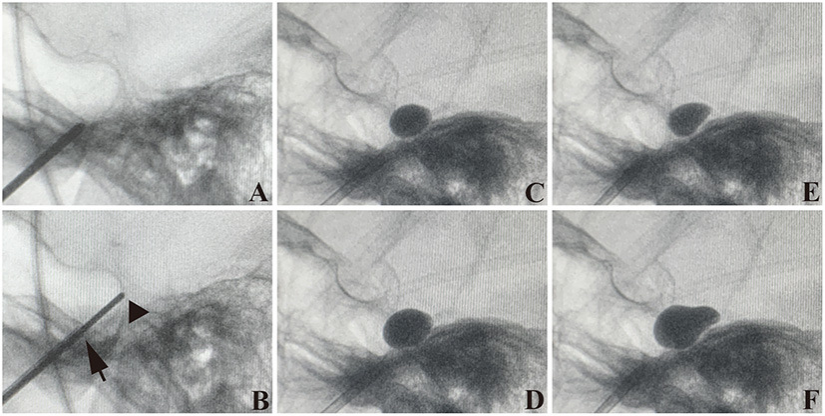
Puncture process. **(A)** Trocar (with stylet in) goes near the foramen ovale (FO). **(B)** The tip of the cannula is at the FO (arrow) but does not penetrate. The tip of the wider needle comes across the clivus on the projection (triangle). **(C,E)** A small 1.5 mL contrast injection to test the proper position, **(E)** is likely a pear. **(D,F)** Enlargement of the balloon **(C,E)**. **(D)** is not a pear. **(F)** is a perfect pear. **(C–F)** were from one PBC surgery.

The entry point is 2 cm from the oral commissure. When puncturing, the foramen ovale (FO) is flet as a soft spot in the bone (Figure 2A). Following stylet withdrawal, the cannulation is performed with a thinner guiding stylet, followed by a larger guiding stylet (Figure 2B). A popping sensation occur during cannulation in most cases. When the cannula tip is positioned at FO, we should avoid pushing it to extend any further, but the guiding stylet should extend slightly further, <1.5 cm (the less the better), and should not extend beyond the clival plane on lateral projection.

After cannulation, we insert a no. 4 Fogarty catheter into the cannula, extending 1.5 cm out of the tip. Before ensuring that the balloon is in the final position of Meckel's cave (MC), we use a 0.15 to 0.2 mL balloon volume projection to determine the proper position (Figures 2C,E). According to intraoperative pressure and the balloon's shape, the final balloon volume ranges between 0.35 and 0.5 mL (Figures 2D,F), with a mean value of 0.4 mL.

### Re-puncturation

If the operation described above failed and no pear shape was observed, we would do re-puncturation. We would choose a more lateral entry point (once only) on the face or a more tilt-up (or otherwise possible) entry angle. Because of the elasticity of the facial skin, the same entry point can use many entry angles. After several re-puncturations, FO may feel like an empty hole; we would try to find a spot around FO's margin with more tension and then push forward the trocar to continue the following procedure.

Another possibility is leaving the cannula at FO and using the thinner guiding needle to investigate the direction with more resistance. Substitution of the operator may also be beneficial. There are three surgeons involved in the procedure. Radiographs should be taken to monitor the whole process. After attempting every possible route and the whole puncturing trajectory may feel like nothing on the way, “persistent elliptical balloon shapes should raise consideration of aborting the procedure (5).”

### Image processing

The figures were processed or drawn using Photoshop and Adobe Illustrator. Whether the pears were good or not was re-evaluated by experienced physicians using radiographs.

### Follow-up

Following PBC surgery, 71 cases were followed up by telephone interviews or outpatient clinic visits. Pain relief was defined as the absence of trigeminal pain in patients who were not on medication.

## Results

### Puncturing and pears or not

**Figure 3:**
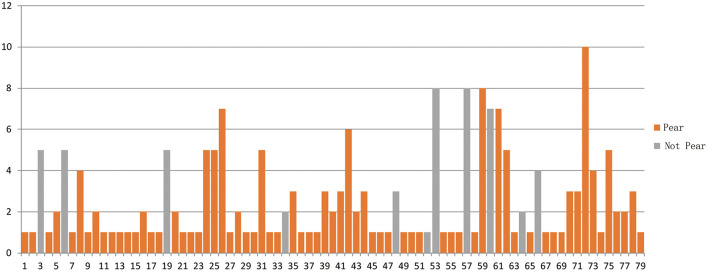
A chronological series of PBC surgery. Horizontal axis shows 79 cases. Vertical axis is puncturing times. Pears or not was retrospectively analyzed, so there was case only got once puncture, but not pears (52).

In chronological sequence, 79 patients underwent PBC procedures. Of those, 50% obtained satisfactory pears in the first puncture, and 35% obtained satisfactory pears after re-puncturation and adjustments (Figure 3). In total, 85% of patients received pears during a single PBC surgery. A total of 68 cases obtained standard pears, of which 40 cases obtained pears upon the first attempt and 28 obtained pears after adjustments.

### Side effects and complications

Table 2 presents side effects and complications. Of 79 cases, 71 were successfully followed up. Facial numbness was the most prevalent complication. We employed four scales: None, Mild, Moderate, and Bothersome, to quantify the numbness from 0 to 3. The average of post-PBC numbness scales of one-puncture cases (39) and multiple punctures (22) were 1.36 and 1.5, respectively, demonstrating no statistically significant difference (*t*-test, *p* = 0.3241). There were limited cases of mastication weakness and hemihypogeusia. The mastication weakness was not significantly different between the two groups, but hemihypogeusia was reported significantly more in multiple punctures cases (*p* < 0.05).

**Table 2:** Side effects and complications for one puncture and multiple punctures.

**Side effects and complications**	**One puncture (*n* = 39)**	**Multiple punctures (*n* = 22)**	***P*** **-value**
Facial numbness score (average ± SE)	1.36 ± 0.029	1.5 ± 0.049	0.3241^a^
Mastication weakness (*n*)	4	0	0.2867^b^
Hemihypogeusia (*n*)	2	6	0.01388^c^

a t-test.

b Fisher exact test.

c Chi-square test.

Facial numbness was quantified from 0 to 3. Thirty-nine patients had one puncture and 22 patients had multiple punctures.

### Outcome

**Figure 4:**
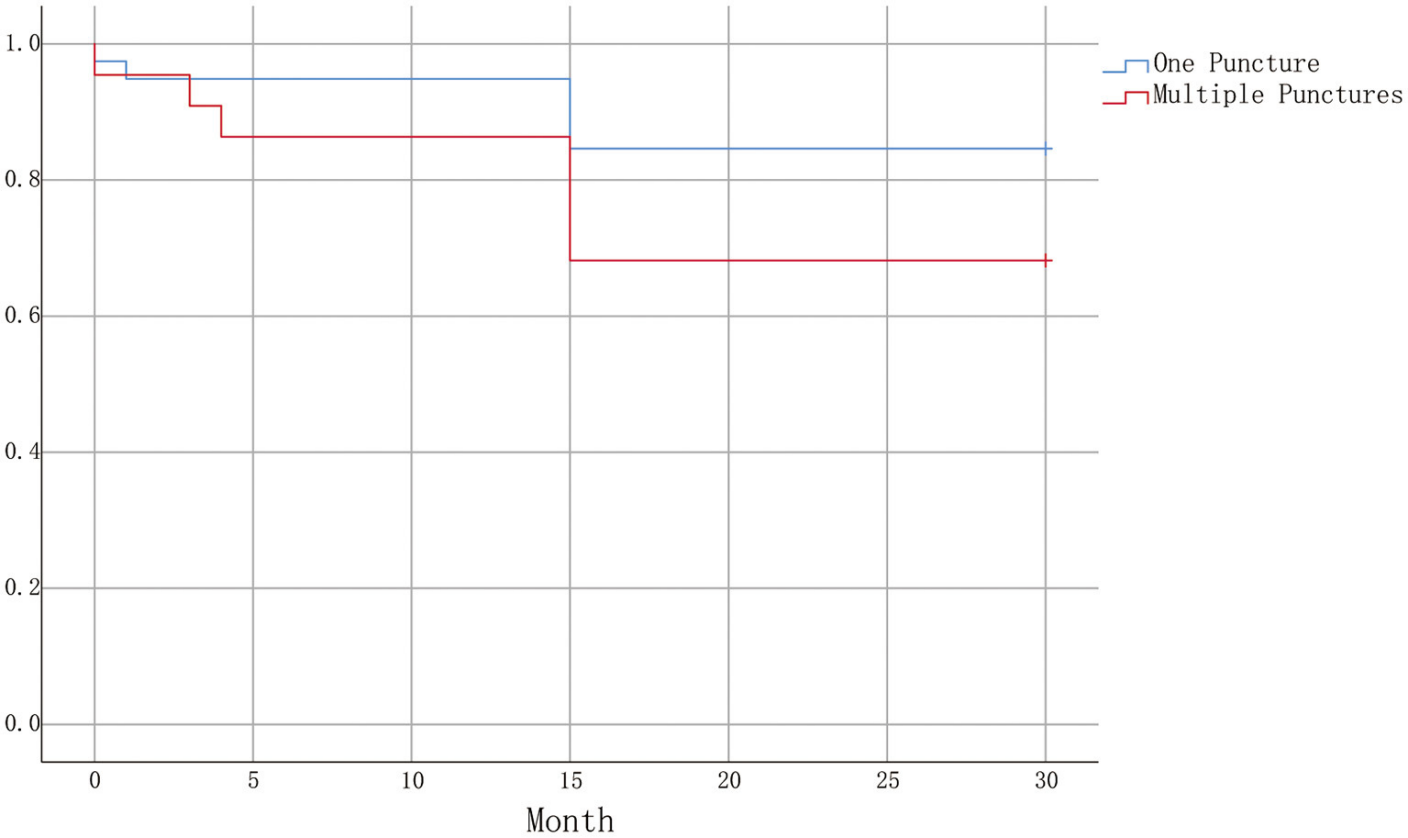
Kaplan-Meier plot illustrating survival curves for one puncture and multiple punctures. A statistically significant difference was not obtained (Log-Rank test *P* = 0.129).

Of patients who obtained good pears, 61 cases were successfully followed up over 30 months. Figure 4 illustrates Kaplan-Meier of one puncture (39 cases) vs. multiple punctures (22 cases). The pain-free rate after 30 months was 84.6% (*n* = 33) for one puncture and 68.2% (*n* = 15) for multiple punctures. Log-Rank test revealed no statistically significant differences between the two groups (*p* = 0.129).

## Discussion

Since PBC surgery's inception, neurosurgeons have attempted to generate a pear-shaped balloon (2, 4, 9). Until now, it may have been the only factor influencing the outcome of PBC in TN treatment (5). The percutaneous approach to FO has long been introduced before Mullan's description of PBC technique. As a result, the safety of PBC procedures might not be the priority that PBC surgeons would consider. The successful rate of obtaining a pear shape varies greatly in different centers from 29.5 to 75% (3, 5, 10). While the criteria of a pear shape may differ for different centers, a pear shape balloon is not a guarantee for all PBC surgeries. According to this study, re-puncturation could result in an additional 35% more pear shapes, implying that the procedure's safety should be reconsidered.

Vascular complications are very uncommon in PBC patients. It is not very likely that damage to extracranial vessels causes serious problems. As for the intracranial part, there are few reports on intracranial hemorrhage and even fewer vascular disorders after percutaneous approach to FO (11–15). The injury of blood vessels around MC appears highly likely to result in disasters. There is pericavernous venous plexus that surrounds mandibular nerves in FO region (16). Although the maxillary nerve does not run along the cavernous sinus's lateral wall, the intracranial extension of Hartel's pathway (from FO to porus trigeminus) is extremely close to the cavernous sinus's posterior wall and may intersect with it at porus trigeminus. Nearly 85% of carotid arteries are exposed under some portion of Meckel's cave and the trigeminal nerve, with only dura and no bone, separating the nerve from the artery (16). Although the trajectory of PBC puncture is surrounded by intracranial blood vessels, there have been almost no intracranial hemorrhage cases following PBC, and sporadic cases of carotid-cavernous fistula following PBC, which are typically labeled as “rare”.

The reason for the relatively low risk of intracranial vascular complications remains unknown. The blunted puncture kits may be beneficial in avoiding damage to large blood vessels. Sometimes, after puncturing and withdrawing the stylet, we could observe venous blood coming out of the cannula, but it would always stop automatically following balloon inflation. We hypothesize that mild bleeding will be stopped by the coagulation system, whereas moderate bleeding will be stopped by the pressure supplied by the inflated balloon. Alvernia et al. (15) summarized some of the anatomical risks associated with percutaneous approach to FO. They indicated that FO approach entails risks, but they only emphasized on the risk of the variant course associated with the extracranial maxillary artery. Although the precise route of PBC puncturing appears to avoid critical intracranial vessels, image-guided techniques and detailed anatomical expertise are required. Our case series revealed no intracranial hemorrhage or vascular disorder, corroborating the evidence that percutaneous approach to FO is safe (even repeated on the same patient).

Nearly all PBC complications are mild, and in contrast to pre-PBC devastating pain, post-PBC side effects are far more tolerable for patients. Facial numbness is the most common complication following PBC. Some physicians believe that post-PBC facial numbness is an indicator of a good outcome. There were very few cases of mastication weakness. Hemihypogeusia has not been previously reported. Interestingly, the trigeminal nerve actually does not have any fibers that provide special sensations (taste). Is it possible that sensation of the food's texture enhances its taste? Without a doubt, repeated punctures caused significantly more damage to the third branch.

Urculo et al. demonstrated that a pear shape appears in the lateral radiographic picture only if a distended balloon is placed in a proper position in MC (4). The effort of each re-puncturation and adjustment is to ensure that the balloon end of catheter remains in MC. To obtain a pear shape, the entry point of catheter to MC must be close to the point where the maxillary nerve joins the ganglion; neither halfway of the route (from FO to porus trigeminus) nor close to the porus trigeminus can do.

It is inevitable that the dural structure and connecting tissue will detach from FO, causing FO to feel empty and making pear acquisition more difficult. To accomplish a pear, we attempted to identify a new percutaneous entry point on the skin, a new entry point at FO, or explore the direction using a thinner guiding needle. Additional 35% pears, with no serious complications and the same outcome with one-time success pears, will compensate for the efforts.

## Conclusions

After failing to get a distinct pear shape, re-puncturation is a good option in PBC. Re-puncturation and adjustment could obtain additional pears with no serious complications. The outcome of pears from this procedure was the same with one-time success pears.

## Data availability statement

The original contributions presented in the study are included in the article/supplementary material, further inquiries can be directed to the corresponding author/s.

## Ethics statement

The studies involving human participants were reviewed and approved by Institutional Review Board of Hangzhou First People's Hospital, China. The patients/participants provided their written informed consent to participate in this study.

## Author contributions

All authors listed have made a substantial, direct, and intellectual contribution to the work and approved it for publication.

## Funding

This research was supported by Medical Health Science and Technology Project of Zhejiang Provincial Health Commission (2021KY229).

## Conflict of interest

The authors declare that the research was conducted in the absence of any commercial or financial relationships that could be construed as a potential conflict of interest.

## Publisher's note

All claims expressed in this article are solely those of the authors and do not necessarily represent those of their affiliated organizations, or those of the publisher, the editors and the reviewers. Any product that may be evaluated in this article, or claim that may be made by its manufacturer, is not guaranteed or endorsed by the publisher.
